# Manufacturing parameter analysis for alumina coating on steel substrate by automated image processing of isolated splats samples

**DOI:** 10.1371/journal.pone.0240928

**Published:** 2020-10-29

**Authors:** N. González, J. Zapata, V. Martínez, R. Gadow, J. García

**Affiliations:** 1 Departamento de Ingeniería Mecánica, Universidad Polite´cnica de Cartagena, Cartagena, Spain; 2 Departamento de Electrónica, Tecnología de Computadoras y Proyectos, Universidad Politécnica de Cartagena, Cartagena, Spain; 3 Institute for Manufacturing Technologies of Ceramic Components and Composites (IMCCC), University of Stuttgart, Stuttgart, Germany; University of Vigo, SPAIN

## Abstract

Thermal spray technology, which involves the Atmospheric Plasma Spraying (APS), encompasses a category of coating processes that supply surface properties to protect or improve the performance of a substrate or component. The coating produced by this technology is built by overlapped splats, whose morphology determines the coating properties. In the same way, the splats obtained in a separated distribution by interposing a perforated mask but using the same thermal spray parameters, has a relationship with the overlapped splats inside the coating. The samples with isolated splats have the advantage of being faster and cheaper to generate and analyse. This article analyses alumina plasma-sprayed splats on steel substrates by image processing techniques, which recognize individual splats and their corresponding morphology (doughnut and pancake) parameters. These parameters allow the user to efficiently classify the splats. After that classification, a quality control can be implemented by comparison between the original and checked sample of isolated splats and a new sample obtained during a small interruption in the normal operation. Additionally, these parameters obtained in an automated way can be used to evaluate the effect of different selections of spraying process parameters.

## Introduction

In thermal spraying, flying molten particles flatten and solidify on a substrate, the so-called splats form lamellae. These technical processes are typically grouped into three major categories: flame spray, electric, and plasma spray. A version of the last type is used in the manufacturing of the Atmospheric Plasma Spraying (APS) coatings, which are usually applied in order to provide specific surface properties and protection from corrosion, erosion and heat [1–5].

The most important parameters to be considered in the assessment of a coating are the adhesion strength and residual stresses, which depend strongly on the splat morphology [6], which, in self, is defined mainly by the properties of the powder, the impact characteristics, solidification, and the substrate conditions. These latter elements are determined by powder shape and size, temperature and velocity of droplets, and by substrate roughness and preheating.

As far as splat solidification on the substrate is concerned, the conditions that affect this phenomenon are: 1. the oxidation, phase, and size, which are characteristics of the impacting particles [7–12]; 2. the surface roughness, presence of condensate and adsorbate, wetting ability, thermal contact resistance and temperature, which are thermophysical properties [13–18]. Thus, during splat solidification and cooling, several effects occur. Firstly, the splats shrink, due to the phase transitions, after which solidification and cooling take place. Secondly, the substrate temperature increases, which causes its expansion. These effects generate quenching and tensile solidification stresses in the coating that directly affect the operating lifetime of the coating-substrate system [19]. Thus, the splat morphology is the link between the manufacturing parameters and the behaviour of the materials during the cooling process. On the other hand, the morphology determines the coating adhesion strength [5]. The mentioned morphological parameters therefore represent useful information to improve the coating quality, so a reliable tool to fulfil this task must be developed to characterize the splat morphologies [5–20].

This work applied image processing to isolated alumina splats by APS on a steel substrate as a tool for splat morphology characterization. This tool has been automated in order to obtain useful information for decision making about production of coated pieces more quickly. Therefore, we show that splat morphology assessment, which is related to the coating adhesion, could be carried out through Image Processing Techniques of Scanning Electron Microscope (SEM) from a top view splat image combined with human experts, thus creating a final stage based on computational intelligence [5,21–23].

The preliminary works are discussed in Section 2 in order to identify the main process parameters that characterize the APS coating and their relationship with the resulting splats. Section 3 describes the experimental methodology to produce the isolated splats samples, the acquisition of images by means of SEM, and the application of image processing techniques. After the image processing stage, several morphological parameters of the splat are obtained by an automated process, selecting the equivalent diameter as the parameter to summarize the splat morphology. The proposed methodology is applied to analyse different splat shapes in Section 4, with a subsequent discussion of the results.

Additionally, the equivalent diameter can be represented together with the spraying parameter in order to obtain useful information that can be applied for the optimization of the manufacturing process. The last section presents the main conclusions and future work is discussed.

## APS parametrization and splat morphology

The APS coating characteristics depend on the process parameters of this type of spraying process: electric power, electrodes geometry, plasma stabilization, coating material and the gases mixture. These parameters determine the droplet temperature and velocity [24]. After the impact, the substrate temperature and roughness also have an influence on the flattening and splats solidification [2,25].

The first step of the present work is to obtain digital images of the coating splats by means of the application of an SEM in order to characterize the splat morphology in APS coatings using image processing techniques. These splat morphologies have usually been classified into several groups: disk, fingered, halo, halo fingered, halo doughnut and fragmented [24,26–31], although the majority of splats in real coatings are in the shape of a doughnut or a disk (pancake). Their complex digital representation as a grey-level image can be quantified and simplified in terms of a vector of different geometrical features using an automated procedure. In this way, the automatic image inspection system determines the equivalent diameter, which is the diameter of a circle with the same area as the splat.

This parameter has already been chosen in previous work. Montavon et al. [28] used several shape factors, such as the equivalent diameter, the elongation factor, and the degree of splashing. The elongation factor is the non-circular nature of the selected feature, and the degree of splashing is the magnitude of peripheral material projections from the splats, generated on impact [32]. Li et al. [33,34] studied APS splats at different Reynolds numbers, finding a relationship between this non-dimensional number and the flattening process. Fantassi et al. [35], Trapaga [36] and Madejski [37], among others, established a connection between the dimensionless Reynolds number on powder particles with an initial diameter ratio and the equivalent diameter of the splat. Tran et al. [38] used ImageJ software, a public domain, Java-based image processing program developed at the National Institutes of Health [39], to count the numbers of splats and to calculate their edges, area, Feret’s diameter and circularity. Because of the well-established equivalence splat shapes and coating properties, the causes of splat fragmentation have been studied [40–42].

On the other hand, image processing applied in the quality control of equipment and materials has become a basic requirement in industry due to the increased competition they face [21]. Zapata et al. [22,23] used image processing techniques in order to design a quality control system in welding using Neural Net- works and an Adaptive Network based Fuzzy Inference System. Kulkarni et al. [43] used x-ray and neutron-scattering imaging techniques to correlate coating properties with microstructure and processing parameters.

It is our supposition that equivalent diameter is useful in parametrizing the morphology of the splat. This splat morphology is related to the coating process quality. Thus, a quality control system can be established by means of image analysis techniques that enable to decide whether a coating is acceptable or not by a simple and inexpensive analysis of splat morphology from a plate with isolated splats and without having to carrying out destructive testing in the coated piece. Therefore, and from our point of view, an automatic inspection system of SEM images of coatings should be divided into the following stages: digitalization of the coating surface, image pre-processing seeking mainly to improve contrast improvement and to denoise, segmenting the scene to isolate the areas of interest and extract features of splats in terms of individual characteristics.

## Experimental methodology

### Spray process and operating parameters

In a schematic way, a DC electric arc, which is struck between non-consumable electrodes within a torch, supplies the heat necessary to produce ionized plasma gas at a high temperature from a flow of inert gases. The coating material, in powder form, is carried in an inert gas stream into the plasma jet, where it becomes molten and is propelled towards the substrate. Therefore, the following parameters should be considered in order to define the spray process: Ar/H2 gas flow and electric current for the plasma gas, the carrier gas and powder mass flow, the powder size, the torch velocity and the substrate temperature.

The gas flow has a strong influence on the temperature for the spray and, obviously, in the plasma flow. Moreover, the ratio between Argon (Ar) and Hydrogen (H2) in the gases has an influence on the process energy. The larger the ratio of H2 is, the bigger the spray energy will be. The value of 40 l/min of Ar is chosen as a process constant, so the energy control related to the gas flow ratio is obtained by the H2 flow. The powder volume flow provided to the process by a GTV PF 4/3 feeder is controlled by the angular velocity of a disk with a slot. The Ar carrier gas flow must be able to support and inject the powder which is external radial injected in the plasma jet after the nozzle exit of the GTV F6 plasma torch. The usual value for the disk angular velocity is 1 rpm, equivalent to a powder flow of 8.5 g/min, whilst for the Ar flow the usual value is 5 l/min.

The powder used in this work, supplied by Ceram GmbH Ingenieurkeramik, was blocky and with a particle size distribution of -20+5 μm. This means that D10 and D90 values are 5 μm and 20 μm, respectively.

The flow velocity at the plasma torch nozzle bears a relation with the performance of the manufacturing process. A high velocity involves the use of a high current and high residual stresses. A common value is 500 mm/s. The temperature of the steel substrate has a strong influence on the splat shape. A temperature of 20°C, lower than normal, was chosen with the aim of producing more complex splat shapes. In the present work we evaluated and parametrized splats from two powder sizes using two sets of operational parameters: current intensity and H_2_ gas flow.

**Fig 1:**
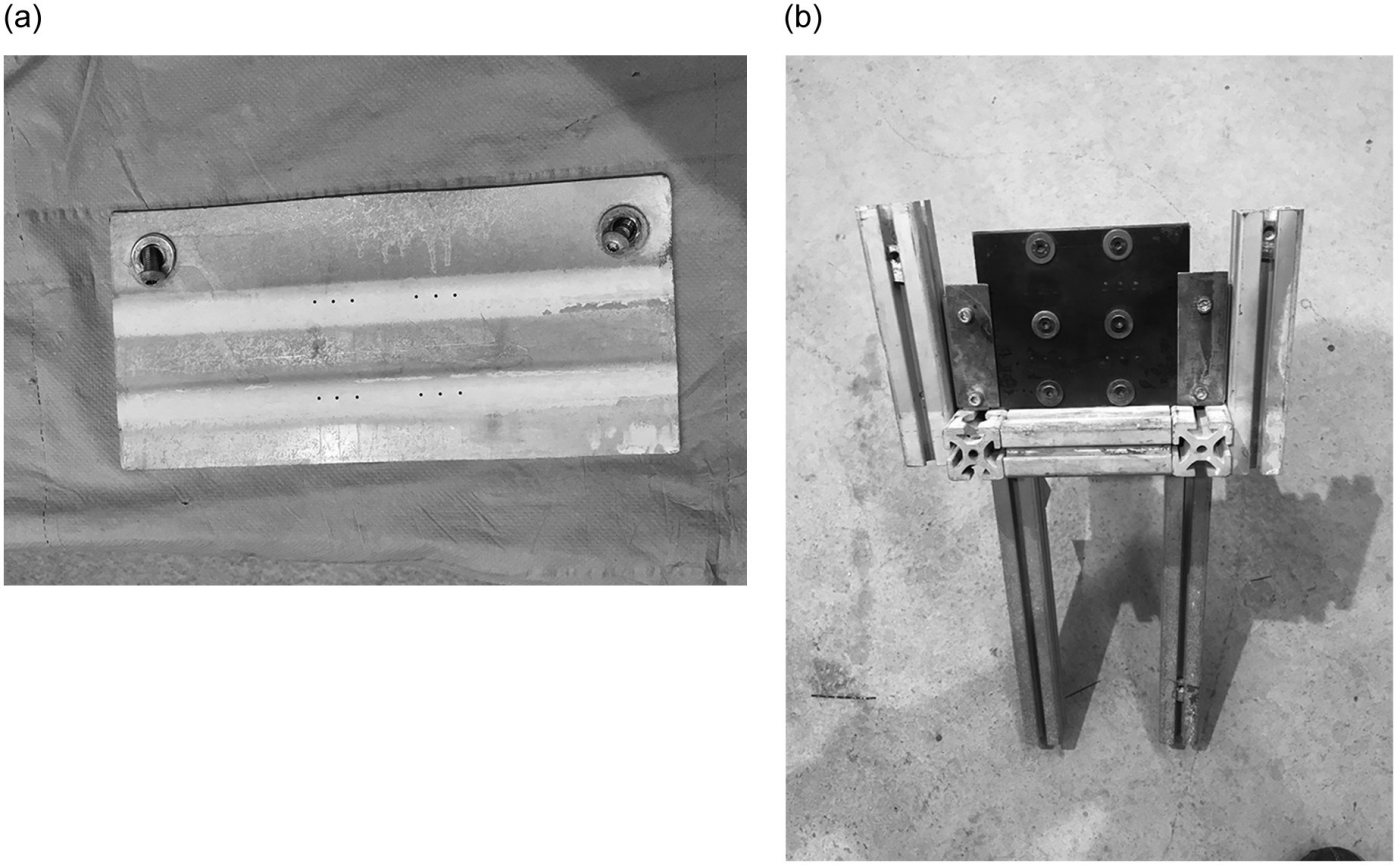
Mask used (a) and support structure (b).

**Fig 2:**
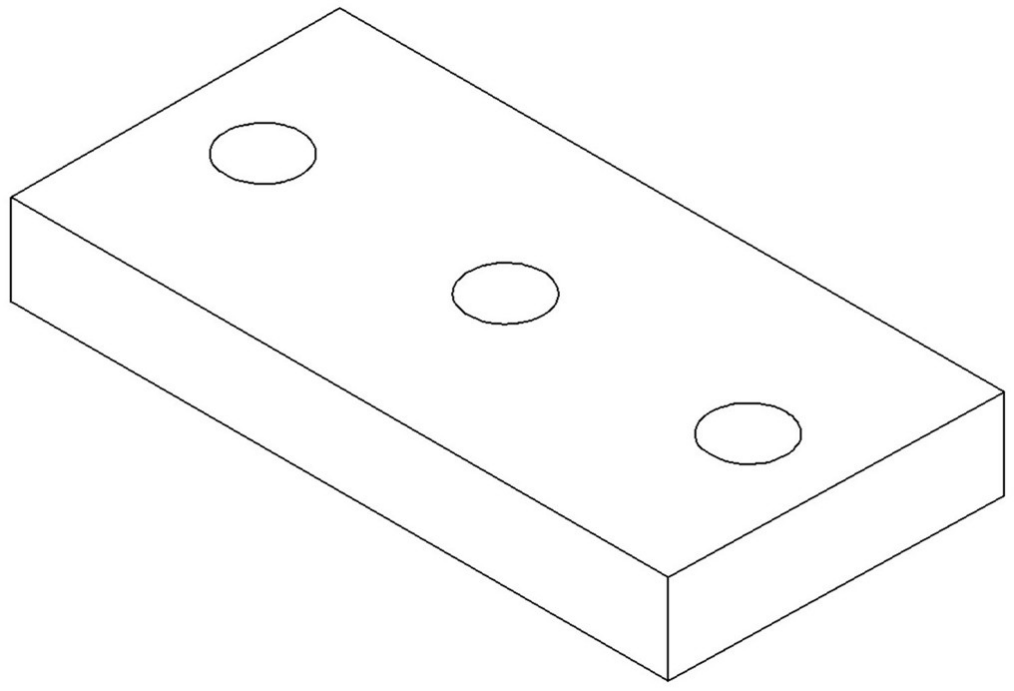
Sample face with three circles.

An experimental procedure to obtain isolated splats was implemented. In this procedure, a mask was used in order to collect individual splats. As shown in Fig 1, the mask had four groups of three holes which were 1 mm in diameter. Each hole provided a circle with splats over a plate which had to be cut to obtain the samples, as shown in Fig 2. Each sample had splats on both faces, in order to make its handling easier with three circles per face. After the sample had been cut, splat images were acquired by an SEM. However, the mask was not used in sample 5; thus, splats spread on the whole faces: A and B. Alumina was shot with different parameters onto each face.

### Image acquisition

The splats were examined by SEM HITACHI, S3500N, and the acquired images had 800×600 pixels with 8 bits per pixel which was adequate for the purposes of this paper. The system recognizes the information in the SEM image related to the actual size of the object, useful to transform from pixels to μm. Several authors [2,5,41,44–46] studied SEM images of splats in order to analyse their morphology, due to the importance of the shape of the splat when assessing the coating and the influence of the coating process parameters. Thus, in the next step, the image processing stage, a vector should be obtained with its components based on splat morphological features: area, perimeter, circularity, solidity, eccentricity, bounding box, Feret’s diameter and equivalent diameter.

The area is therefore obtained counting the pixels in the region. The perimeter is calculated considering adjoining pairs of pixels around the edge of the region and is measured by means of the distance between each adjoining pair of pixels around the edge of the region. The circularity is calculated from the former geometrical parameters. The solidity indicates the ratio between the areas of the splat and the convex hull (smallest polygon) which can contain that splat. The splat eccentricity represents the value of this parameter for an ellipse with equal second moments. The Feret’s diameter is calculated by means of a bounding box of the splat.

### Image processing stages

**Fig 3:**
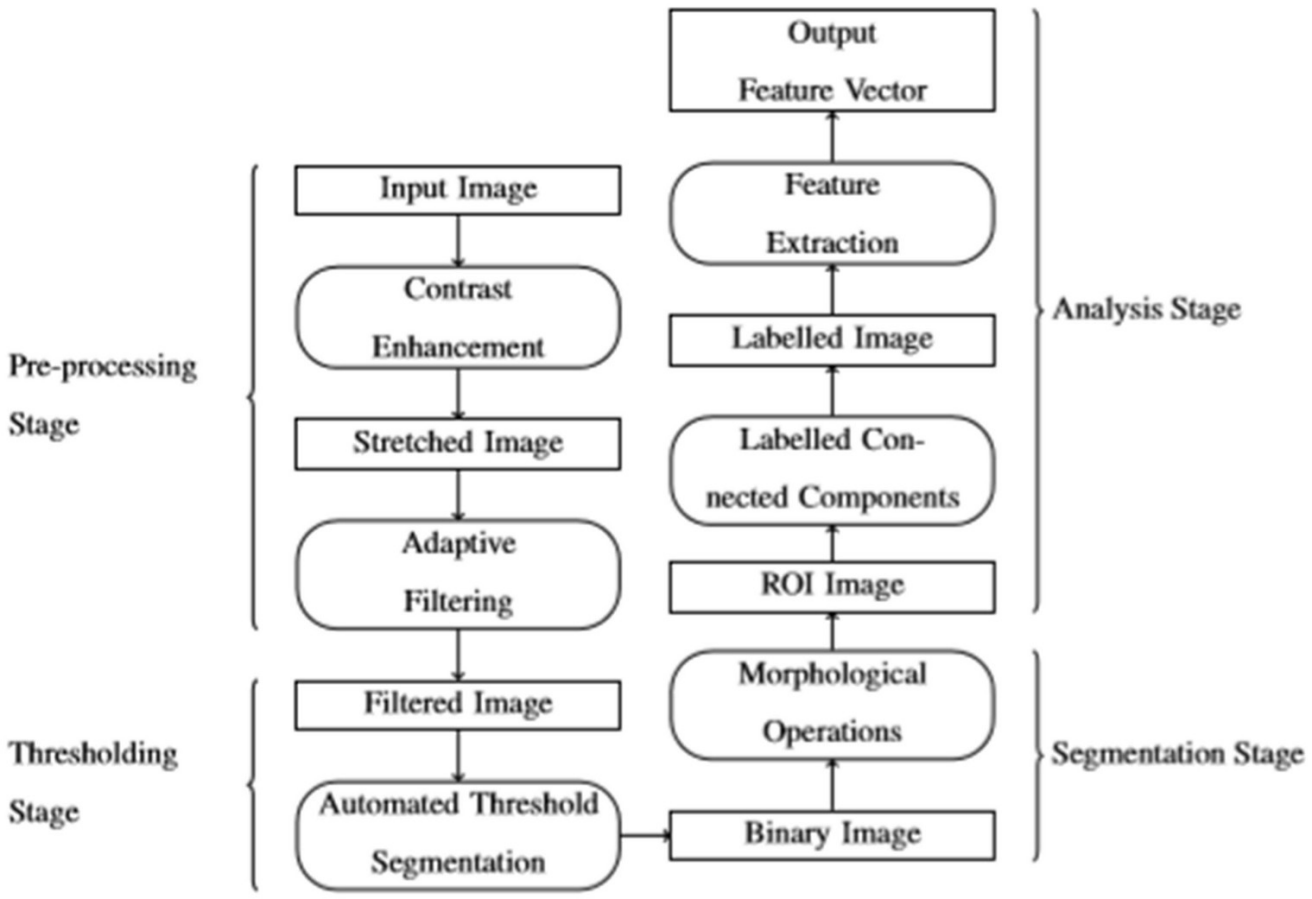
Automatic image processing procedure to obtain discriminatory features to simplify the image scene.

The original complex image will be simplified by Image Processing techniques in a step-by-step way. Fig 3 shows the four stages of the procedure.

The first step or pre-processing stage is based on two pre-processing procedures: contrast enhancement and image filtering, in order to provide a better dynamic range and remove noise and possible artefacts. The first procedure stretches the intensity value of the previous image into a new image, which transforms the values of pixels throughout the range of the histogram. On the other hand, the most important noise in the images from the microscope, to be more precise, is due to the secondary electron detector, and has a homogeneous distribution over the image with intensity differences between contiguous pixels [47]. The second procedure uses two filter types in order to reduce any noise: an adaptive Wiener, and a 3-by-3 Gaussian low pass.

In the second step or thresholding stage, the resulting image from the previous step is transformed from a normalized grey level coded from black or 0 background and white or 1 foreground with the aim of obtaining a binary image and marking the boundaries of objects located in the background. In our case, in order to obtain a binary image, we needed to obtain a bi-level thresholding with an overall threshold. The procedure used Otsu’s method [48], which chooses the threshold to minimize the intraclass variance of the black and white pixels. Our second goal is obtained directly because a binary image with objects in its background has the borders of these objects delimited because boundaries are obtained with the cross between binary pixels (black and white).

For the third step or segmentation stage, the former image is submitted to different morphological operations, which applies a structuring element to the image obtained in the second step. The procedure executes two opposing algorithms: opening and closing image algorithms. The opening algorithm performs the morphological opening on the greyscale or binary image by an erosion followed by a dilation, using the same structuring element for both operations. The morphological close operation is a dilation followed by an erosion.

This simple structuring element (a kernel) is itself a binary image, which in our case is a rectangle (5×5). In dilation, as the kernel is scanned over the image, the algorithm computes the maximal pixel value overlapped by the kernel and replaces the image pixel with that maximal value. As can be expected this maximizing operation causes bright regions within an image to “grow” thus the name dilation. Erosion is the sister of dilation and computes a local minimum over the kernel area. With this step, splat boundaries are closed and smoothed, and artefacts near to the boundaries or inside the splat are removed.

Until now, our image is no more than a background with white pixels (no objects) on it. We must cluster those white pixels conforming an object. The labelling technique is based on connect components algorithm which is applied to the resulting image for the following step, following the same procedure as Haralick and Shapiro [49]. Object (or blob) extraction is generally performed on the resulting binary image from a thresholding step, but it can also be applicable to greyscale and colour images. We use four connected background neighbours algorithm for that task, which considers that contiguous pixels connected along the vertical or horizontal direction are part of the same object splat. Also, for binary images, connected background pixels 0’s delimits the objects boundaries connected foreground pixels 1’s. As a result, a matrix equivalent to the original image associates label 0 to pixels that belong to the background, label 1 for pixels in one object, label 2 for pixel in a second object and so on.

**Fig 4:**
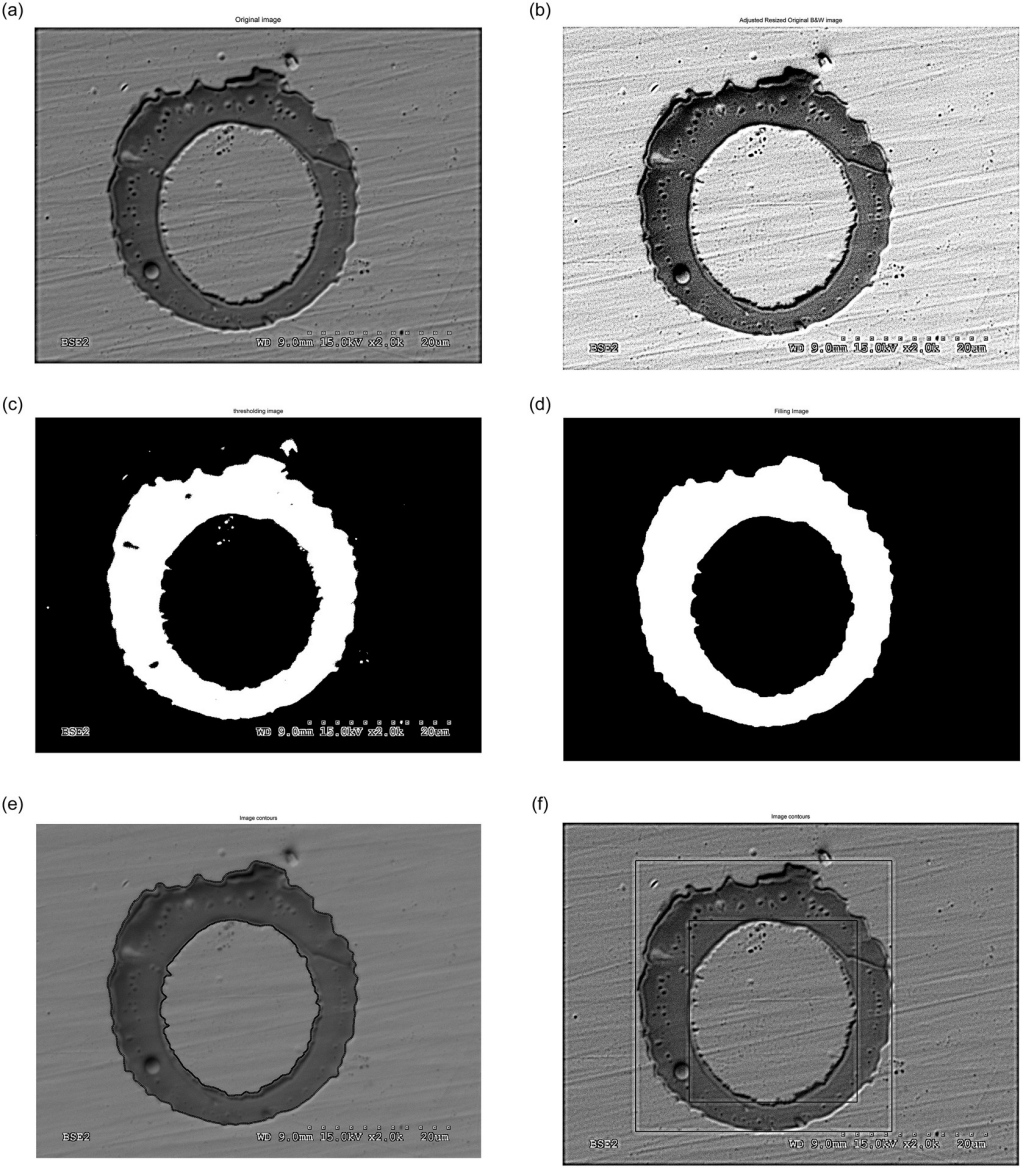
Procedure output images. Original image (a), adjusted resized image (b), thresholding image (c), filling image (d), superposing polygon fitting (e), and superposing bounding boxes (f).

**Fig 5:**
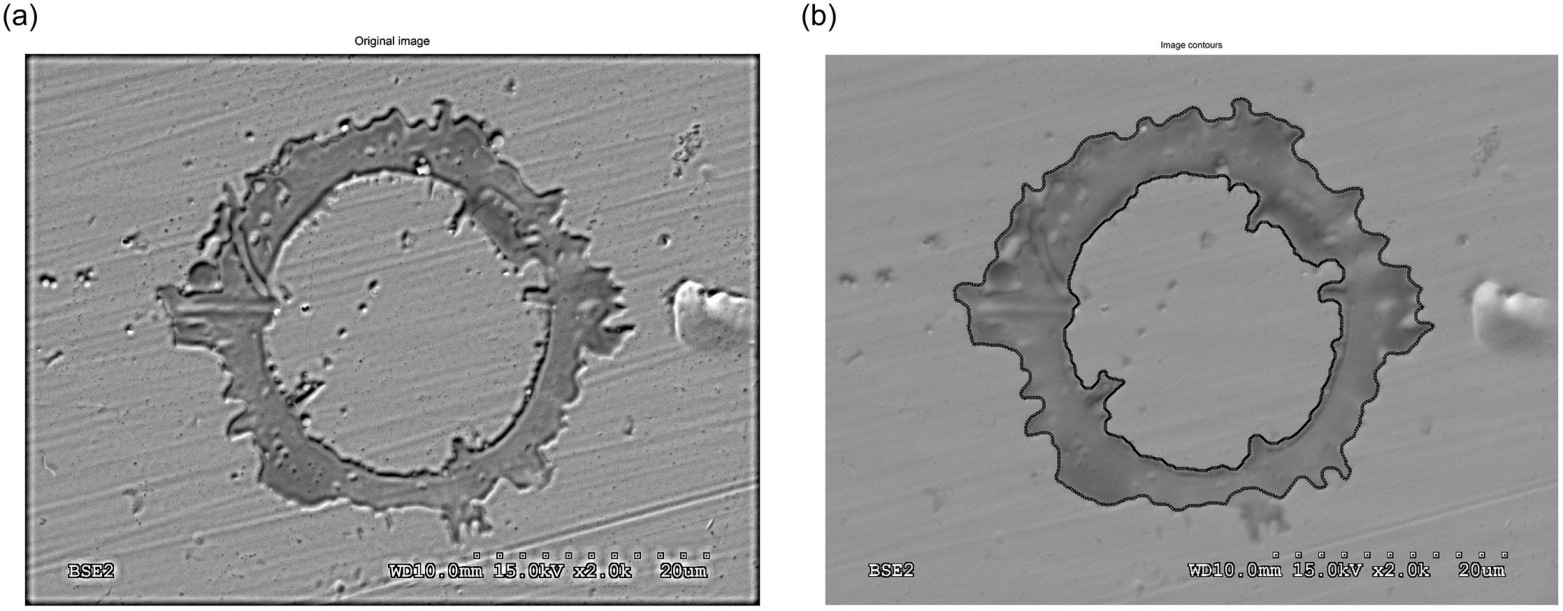
Original image (a) and its superposing polygon fitting (b).

After that, the shape descriptors or morphological properties of all the labelled objects are calculated. However, real splats and tiny fragments must be differentiated. Any object which consists of only a few pixels is considered an artefact and not a splat. Needless to say, in order to make this differentiation we must establish a threshold which allows us to classify objects with an area lower than it as fragments. To find this limited threshold the alumina flattening ratio used by Li is selected [50]. That ratio establishes a relationship between the powder size and the splat diameter. Therefore, if the powder size is over 5 μm we can expect a splat diameter over 13.8 μm, which means a surface area of 150 μm^2^. The zones classified as fragments will be considered to calculate the fragment ratio. The detected objects are represented by a number according to the corresponding label of their pixels. Some output images from the different stages in Fig 3 are shown in Fig 4 as an example of the procedure used. Once we have cleared all the tiny artefacts, their morphological properties are obtained properly. The superposing polygon fitting for other splats is shown in Fig 5.

### Equivalent diameter calculation

**Fig 6:**
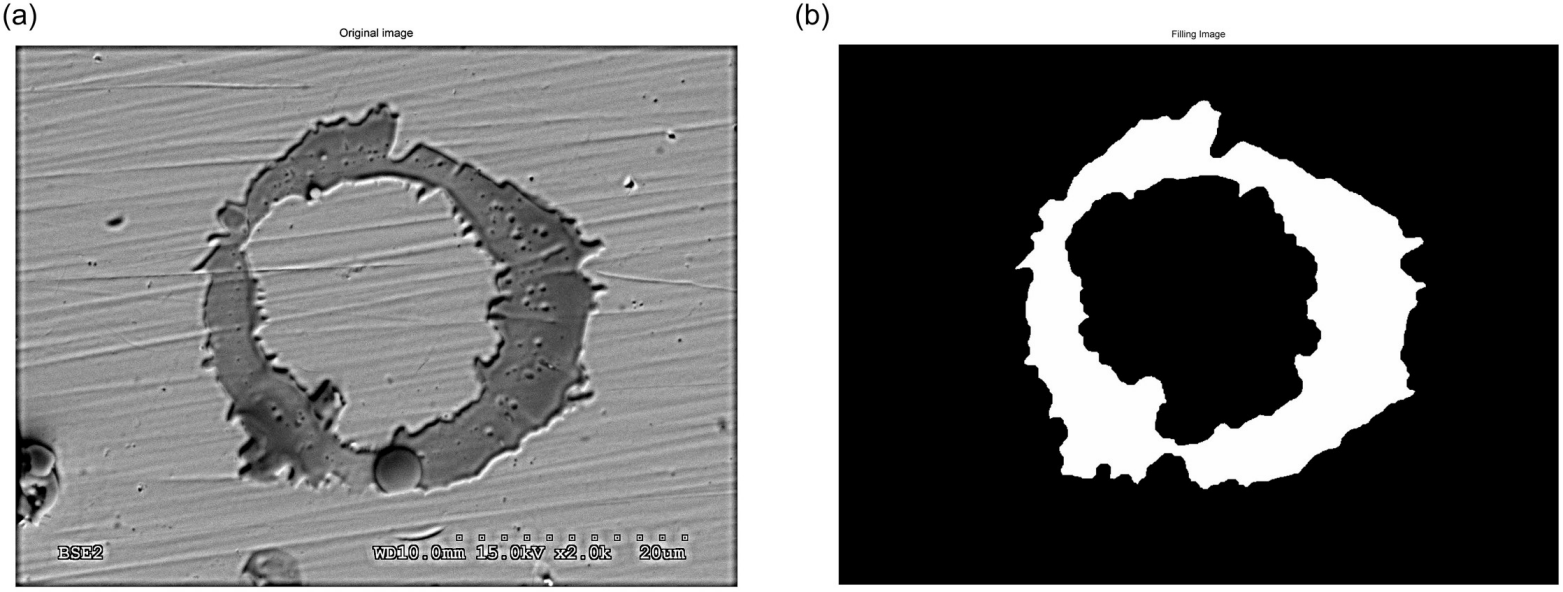
Input and output image after applying our procedure. Original image (a); filling image (b).

The equivalent diameter is a measure of an object’s size. As is shown in Fig 6, we selected an image with several complex elements (a splat with a doughnut shape and different objects and particles on the image), and applied the aforementioned procedure on this SEM image, obtaining a simpler labelled image. This simple image is formed with two labels: the background is labelled with 0 and the foreground with 1.

**Fig 7:**
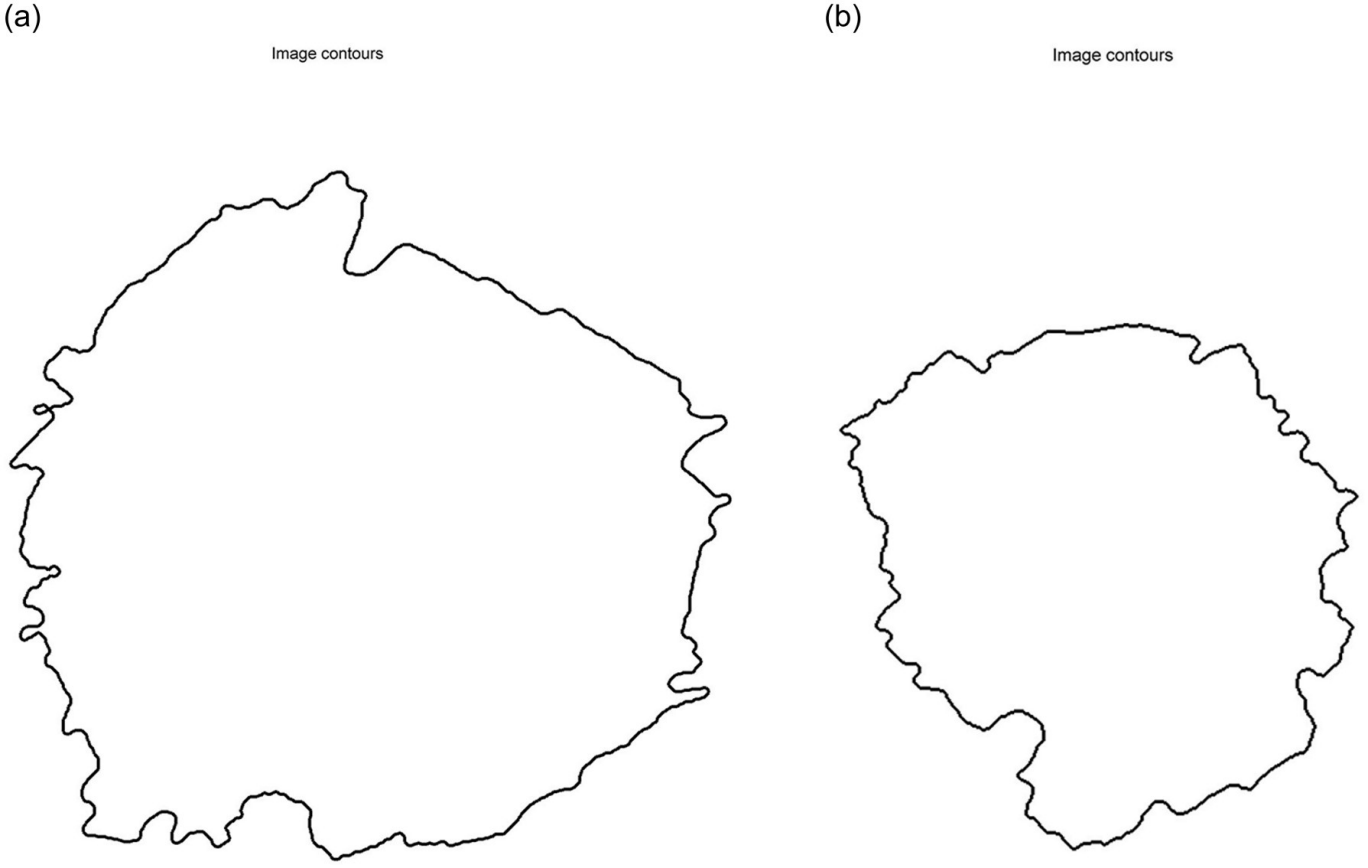
Outer (a) and inner (b) edge of splat.

For the case of a doughnut splat, two equivalent diameters are calculated: the equivalent diameter of the hole of the doughnut (inner contour) and the equivalent diameter of the outer contour. It is possible to remove the hole of the doughnut because the image is a labelled image, as is shown in Fig 7. In a labelled image we can know if a pixel labelled as background (0 or black) is inside or outside of a limited object where its pixels are labelled as foreground (1 or white). Finally, both equivalent diameters for the outer contour and inner contour are available and can be used to classify the splats. On the other hand, pancake splats have outer equivalent diameters. This same operation is used for other previously mentioned geometrical features (e.g., bounding box, enclosing ellipses, polygon fitting, etc).

All these operations provide values in pixels, which must be transformed into μm. The SEM image width in mm (L’) and the SEM scale in mm (S’) can be measured, and since the length of the SEM scale in μm (Sr) is provided by the microscope, it is possible to write the following proportion in order to obtain the image width in μm (L):

$$\text{L}=\text{L}’\cdot {\text{S}}_{r}/\text{S}’$$ (1)

This operation should be calculated only once for an SEM image and can be used with the other images keeping the same SEM scale and SEM image width.

The equivalent inner and outer diameters in pixels (dfp) can be transformed into μm (df), by the ratio between the image width measured in pixels (Lp) and in μm (L):

$${\text{d}}_{f}={\text{d}}_{\text{f}p}\cdot \text{L}/{\text{L}}_{p}$$ (2)

## Results and discussion

In summary, five APS coating samples were produced at the Institute for Manufacturing Technologies of Ceramic Components and Composites IMCCC using the APS gun GTV type F6. Table 1 shows the parameters used.

**Table 1:** Atmospheric plasma spray process parameters.

Sample	S_1_	S_2_	S_3_	S_4_	S_5A_	S_5B_
**Powder size (μm)**	20	20	40	40	40	40
**Hydrogen mass flow rate (slpm)**	12	8	12	8	12	12
**Current intensity (A)**	600	550	600	550	600	600
**Powder carrier gas mass flow rate (slpm)**	5	5	10	10	10	10
**Angular velocity powder supplier, (rpm)**	1	1	1	1	0.5	1

**Fig 8:**
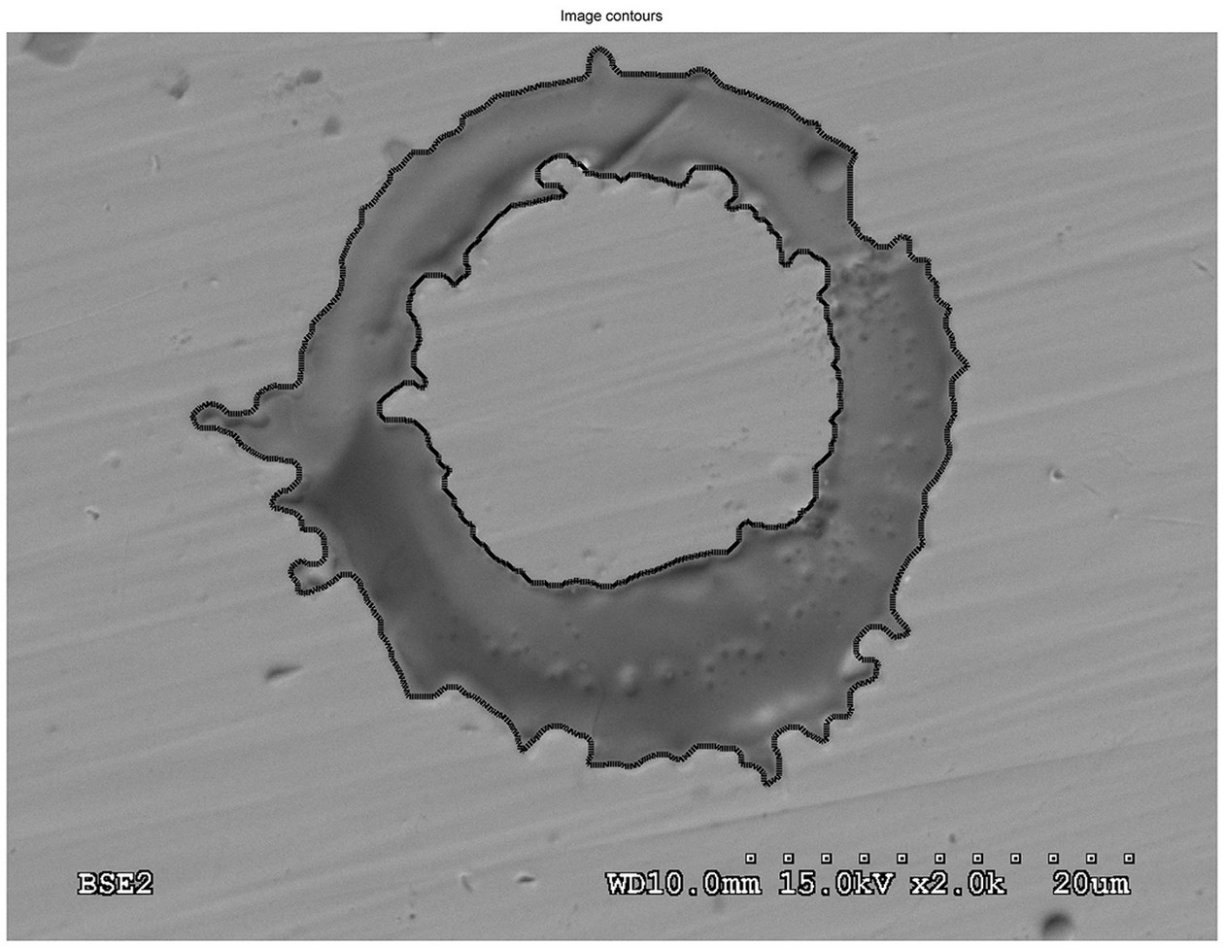
Superposing polygon fitting of a doughnut splat in sample 3.

Fig 8 shows the splats correctly characterized and segmented by the system. To be more precise, the splats were segmented and measured in terms of their size, circularity, perimeter, eccentricity, area, solidity, Feret’s diameter and equivalent diameter in order to simplify the scene. The major splashing and the high fragment ratio are consistent with the tiny droplet on the surface.

Subsequently, the splats of all samples we analysed. In order to explain the analysis process, we used sample 1; the doughnut splat data in pixels are shown in Table 2. In order to obtain these dimensions in μm, the SEM scale length in pixels and in μm is used, see Table 3, where the images width is also given. Using that information and Eqs (1) and (2), the equivalent diameters can be calculated in μm, Table 4. In that table, for example, splat 10 in circle 3 and sample 1 is noted by S1.3 10. Thus, a total of 12 doughnut splats have been analysed, with their equivalent diameters being obtained.

**Table 2:** Doughnut splat diameters in sample 1, in pixels.

Name	Outer dfp (pixel)	Inner dfp (pixel)
**S.1.3_10**	38.794	22.568
**S.1.2_7**	47.620	29.164
**S.1.1_9**	36.772	22.680
**S.1.4_14**	42.054	24.824
**S.1.5_11**	41.120	22.568
**S.1.6_7**	31.895	19.512
**S.1.3_12**	37.797	27.315
**S.1.2_8**	33.833	21.705
**S.1.1_7**	57.007	37.526
**S.1.3_9**	35.396	22.112
**S.1.1_11**	44.338	32.332
**S.1.3_7**	40.022	29.273

**Table 3:** SEM scales in pixels and μm from doughnut splats image in sample 1, with their widths.

Name	SEM scale (pixel)	SEM scale (μm)	Lp (pixel)	L (μm)
**S.1.3_10**	32.0	20	84	82.500
**S.1.2_7**	20.0	10	84	66.000
**S.1.1_9**	32.0	20	84	82.500
**S.1.4_14**	20.0	10	84	66.000
**S.1.5_11**	24.0	10	84	55.000
**S.1.6_7**	33.0	20	84	80.000
**S.1.3_12**	32.0	20	84	82.500
**S.1.2_8**	20.0	10	84	66.000
**S.1.1_7**	20.0	10	136	66.000
**S.1.3_9**	20.5	9	84	57.951
**S.1.1_11**	24.0	10	84	55.000
**S.1.3_7**	24.0	10	84	55.000

**Table 4:** Doughnut splat dimensions in sample 1, in μm.

Name	Outer df (μm)	Inner df (μm)	Thickness (μm)
**S.1.3_10**	38.101	22.165	7.968
**S.1.2_7**	37.415	22.914	7.251
**S.1.1_9**	36.115	22.275	6.920
**S.1.4_14**	33.042	19.505	6.769
**S.1.5_11**	26.924	14.776	6074
**S.1.6_7**	30.377	18.582	5.897
**S.1.3_12**	37.122	26.827	5.147
**S.1.2_8**	26.583	17.054	4.764
**S.1.1_7**	27.663	18.211	4.726
**S.1.3_9**	24.419	15.255	4.582
**S.1.1_11**	29.031	21.170	3.931
**S.1.3_7**	26.205	19.167	3.519

Table 5 shows the pancake splats equivalent diameters in μm in sample 1, obtained following the same procedure used for Table 4.

**Table 5:** Pancake splat diameters in sample 1, in μm.

Name	df (μm)	Name	df (μm)
**S.1.2_1**	45.319	**S.1.5_6**	29.827
**S.1.6_1**	38.321	**S.1.2_2**	29.794
**S.1.3_2**	38.109	**S.1.1_2**	28.541
**S.1.3_4**	36.771	**S.1.4_13**	28.476
**S.1.2_3**	35.294	**S.1.4_4**	27.923
**S.1.4_2**	35.099	**S.1.3_5**	27.807
**S.1.6_2**	35.008	**S.1.4_1**	27.724
**S.1.6_3**	34.260	**S.1.6_5**	27.702
**S.1.6_6**	33.329	**S.1.4_3**	27.193
**S.1.4_9**	33.080	**S.1.3_3**	26.469
**S.1.5_3**	32.126	**S.1.4_6**	26.156
**S.1.2_5**	31.551	**S.1.4_5**	25.992
**S.1.2_4**	31.114	**S.1.6_4**	25.291
**S.1.4_11**	30.507	**S.1.1_3**	24.428
**S.1.4_12**	30.458	**S.1.4_7**	24.422
**S.1.5_10**	30.222	**S.1.1_1**	24.395
**S.1.5_4**	30.041	**S.1.5_2**	22.577

Tables 6 and 7 summarize information about the mean diameter and diameter range for all the samples. Then two relationships can be obtained: i) the splat morphology seems to not be related with the mean equivalent diameter for doughnut and pancake splats; and ii) the circular crown width depends on the mean outer equivalent diameter. The larger the size of the doughnut splat is, the greater the thickness is.

**Table 6:** df of pancake in μm for all the samples.

	Range of df	Mean of df
**S**_**1**_	16.09–38.32	27.41
**S**_**2**_	20.96–23.13	22.05
**S**_**3**_	20.26–83.79	45.46
**S**_**4**_	31.16–51.75	41.45
**S**_**5A**_	30.15–56.95	43.88
**S**_**5B**_	28.76–54.70	44.09

**Table 7:** df and thickness of doughnut in μm for all the samples.

	Range of outer df	Mean of outer df	Thickness range	Thickness average
**S**_**1**_	24.42–38.10	31.71	3.52–9.97	5.63
**S**_**2**_	-	-	-	-
**S**_**3**_	32.98–35.76	34.67	5.24–6.47	5.83
**S**_**4**_	-	43.17	-	7.66
**S**_**5A**_	28.55–53.55	40.55	5.14–13.10	8.46
**S**_**5B**_	26.37–56.46	40.00	5.14–13.10	8.46

**Fig 9:**
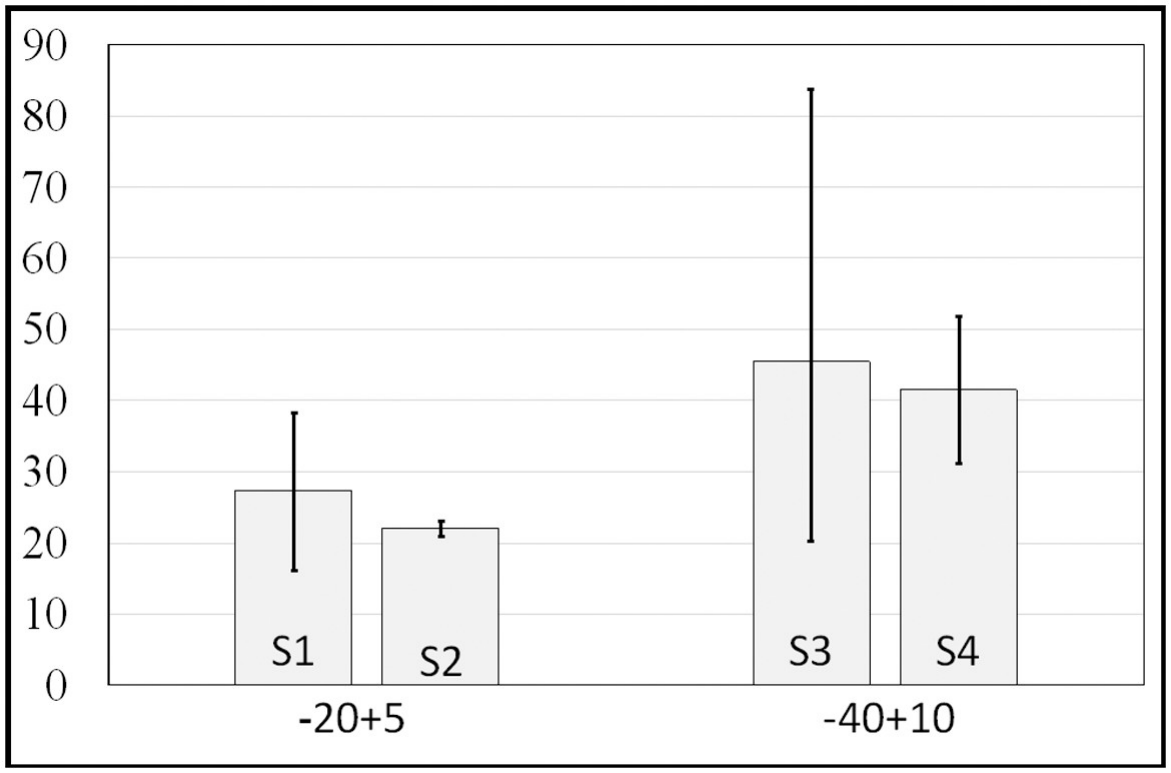
Mean equivalent diameter (μm) for pancake splats vs characteristic powder particle size.

**Fig 10:**
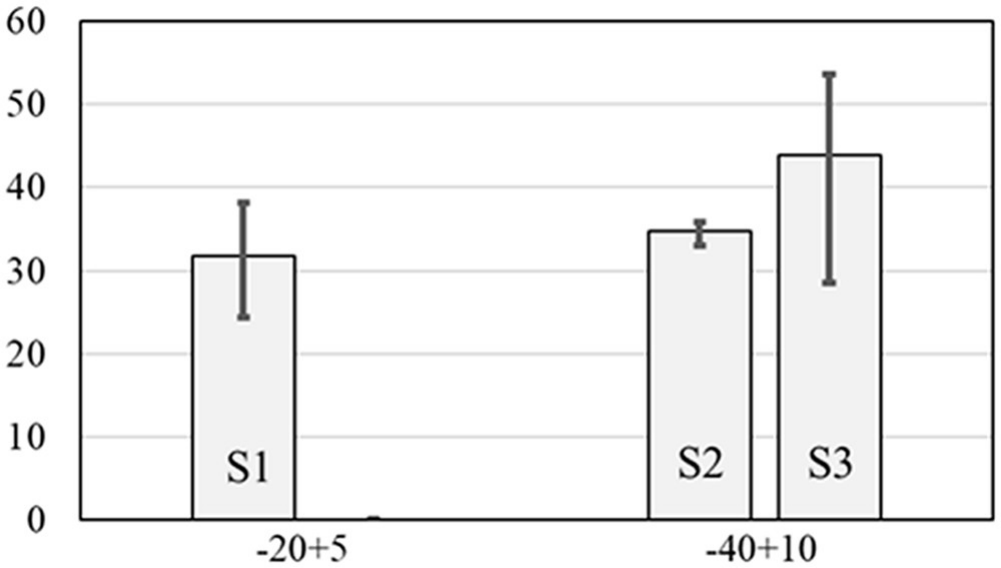
Mean equivalent outer diameter (μm) for doughnut splats vs characteristic powder particle size.

**Fig 11:**
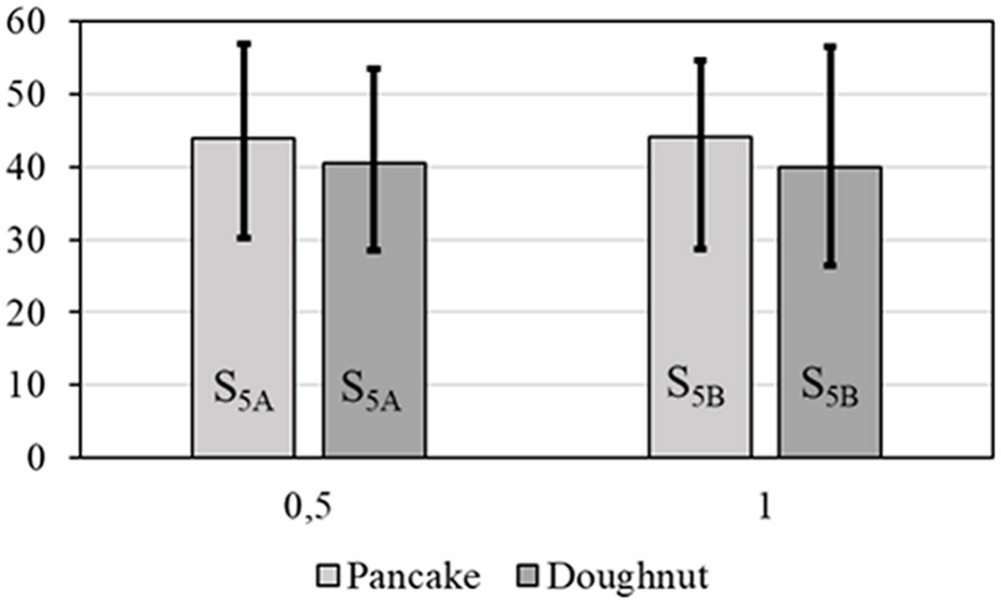
Mean equivalent diameter (μm) vs revolutions per minute.

After the splat parametrization the relationship between the splat morphologies and spraying parameters can be represented. Fig 9 shows the mean equivalent diameter of the pancake splats versus different powder sizes for two sets of current intensity and hydrogen mass flow rate. The larger the powder size is, the bigger the diameter is, and the same relationship is found between current intensity and equivalent diameter. Fig 10 shows the same relation for the doughnut splats. That kind of morphology is less sensitive to the powder size. A comparison between Figs 9 and 10 shows the sizes of the two morphologies to be similar. Fig 11 shows the outer diameters of the pancake and doughnut splats versus different powder-supplier angular velocities. The revolutions per minute has a perceptible influence on the equivalent diameter.

From the results, the sensitivity of the splats parameters from the operation process can be checked.

## Conclusions and future work

This article analyses isolated alumina plasma-sprayed splats on a steel substrate making use of image processing techniques. An experimental procedure to obtain isolated splats joint to an additional analysis in order to classify doughnut and pancake splats by their morphological features was implemented. The comparison between the pancakes and doughnut morphological features obtained under different coating conditions provides a better insight into the splat formation and its relations with the process parameters.

Equivalent diameter among other morphological features is obtained by automatic procedure, which is tested with numerous splat images. The splat characterization is possible after a correct segmentation implemented by the procedure. To be more precise, the splats were segmented and measured morphologically. Therefore, the automated procedure applied to isolated splats sample is reliable to be used as a quality control or, at least, as a fast and efficient alternative for the inspection of coated pieces.

Taking all this into account and as future work, the authors are also interested to include other process parameters in the study, as is the case of substrate preheating. This preheating provides very uniform splats morphological features making easier and more reliable the automated parametrization of the isolated splat proposed in this work. With this tool and expert knowledge a more evolved version will be developed, implementing in that way an expert system to acquire SEM images and to decide without human intervention or slower and more expensive test, whether the splats present specific properties, characteristics of an suitable coating. Furthermore, a comparison between different equivalent diameters and adherence values will provide additional information about the relationship between the splat geometries and suitable coatings for specific applications.

## References

[1] Dhiman R., McDonald A. G., Chandra S., Predicting splat morphology in a thermal spray process, Surface and Coatings Technology 201 (18) (2007) 7789–7801. URL 10.1016/j.surfcoat.2007.03.010.

[2] Davis J. R., et al, Handbook of thermal spray technology, ASM international, 2004.

[3] Heimann R. B., Plasma Spray Coating, Wiley-VCH, 2008.

[4] Pawlowski L., The Science and Engineering of Thermal Spray Coatings, John Wiley & Sons, 2008.

[5] Mulero M., Zapata J., Vilar R., Martínez V., Gadow R., Auto- mated image inspection system to quantify thermal spray splat morphology, Surface and Coatings Technology 278 (2015) 1–11. URL http://www.sciencedirect.com/science/article/pii/S0257897215301729.

[6] Okumus S. C., Microstructural and mechanical characterization of plasma sprayed Al2o3–TiO2 composite ceramic coating on Mo/cast iron substrates, Materials Letters 59 (26) (2005) 3214–3220. URL http://www.sciencedirect.com/science/article/pii/S0167577X05005252.

[7] J. Houben, Relationship of the adhesion of plasma sprayed coatings to the process parameters: Size, velocity and heat content of the spray particles, Ph.D. thesis, Technische Universiteit Eindhoven (December 1988).

[8] Syed A., Denoirjean A., Hannoyer B., Fauchais P., Denoirjean P., Khan A., et al, Influence of substrate surface conditions on the plasma sprayed ceramic and metallic particles flattening, Surface and Coatings Technology 200 (7) (2005) 2317–2331. URL 10.1016/j.surfcoat.2005.01.014.

[9] Yin Z., Tao S., Zhou X., Ding C., Particle in-flight behavior and its influence on the microstructure and mechanical properties of plasma-sprayed al2o3 coatings, Journal of the European Ceramic Society 28 (6) (2008) 1143–1148. URL 10.1016/j.jeurceramsoc.2007.09.050.

[10] Zhang C., Li C.-J., Liao H., Planche M.-P., Li C.-X., Coddet C., Effect of in- flight particle velocity on the performance of plasma-sprayed ysz electrolyte coating for solid oxide fuel cells, Surface and Coatings Technology 202 (12) (2008) 2654–2660. URL 10.1016/j.surfcoat.2007.09.037.

[11] Zhang C., Kanta A.-F., Li C.-X., Li C.-J., Planche M.-P., Liao H., et al, Effect of in-flight particle characteristics on the coating properties of atmospheric plasma-sprayed 8molneural networks, Surface and Coatings Technology 204 (4) (2009) 463–469. URL 10.1016/j.surfcoat.2009.08.009.

[12] Mantry S., Jha B., Mandal A., Mishra D., Mishra B., Chakraborty M., Influence of in-flight particle state diagnostics on properties of plasma sprayed ysz-ceo 2 nanocomposite coatings, International Journal of Smart and Nano Materials 5 (3) (2014) 207–216. URL 10.1080/19475411.2014.941041.

[13] Pershin V., Lufitha M., Chandra S., Mostaghimi J., Effect of substrate temperature on adhesion strength of plasma-sprayed nickel coatings, Journal of Thermal Spray Technology 12 (3) (2003) 370–376. URL 10.1361/105996303770348249.

[14] Fukumoto M., Huang Y., Flattening mechanism in thermal sprayed nickel particle impinging on flat substrate surface, Journal of thermal spray technology 8 (3) (1999) 427–432.

[15] McDonald A., Moreau C., Chandra S., Thermal contact resistance between plasma-sprayed particles and flat surfaces, International Journal of Heat and Mass Transfer 50 (9–10) (2007) 1737–1749. URL 10.1016/j.ijheatmasstransfer.2006.10.022.

[16] Jiang X., Wan Y., Herman H., Sampath S., Role of condensates and adsorbates on substrate surface on fragmentation of impinging molten droplets during thermal spray, Thin Solid Films 385 (1–2) (2001) 132–141. URL 10.1016/S0040-6090(01)00769-6.

[17] Tanaka Y., Fukumoto M., Investigation of dominating factors on flattening behavior of plasma sprayed ceramic particles, Surface and Coatings Technology 120–121 (1999) 124–130. URL 10.1016/S0257-8972(99)00348-5.

[18] Y. Tanaka, M. Fukumoto, Influence of solidification and wetting on flattening behavior of plasma sprayed ceramic particles, in: ISAEM 2000: 2nd Inter- national Symposium on Designing, Processing and Properties of Advanced Engineering Materials, 2000, pp. 518–523.

[19] Wenzelburger M., Escribano M., Gadow R., Modeling of thermally sprayed coatings on light metal substrates: layer growth and residual stress formation, Surface and Coatings Technology 180–181 (2004) 429–435. URL 10.1016/j.surfcoat.2003.10.125.

[20] Zhang H., Wang X., Zheng L., Jiang X., Studies of splat morphology and rapid solidification during thermal spraying, International Journal of Heat and Mass Transfer 44 (24) (2001) 4579–4592. URL 10.1016/S0017-9310(01)00109-0.

[21] Vilar R., Zapata J., Ruiz R., An automatic system of classification of weld defects in radiographic images, NDT & E International 42 (5) (2009) 467–476. URL 10.1016/j.ndteint.2009.02.004.

[22] Zapata J., Vilar R., Ruiz R., An adaptive-network-based fuzzy inference sys- tem for classification of welding defects, NDT & E International 43 (3) (2010) 191–199. URL 10.1016/j.ndteint.2009.11.002.

[23] Zapata J., Vilar R., Ruiz R., Performance evaluation of an automatic in- spection system of weld defects in radiographic images based on neuro- classifiers, Expert Systems with Applications 38 (7) (2011) 8812–8824. URL 10.1016/j.eswa.2011.01.092.

[24] Kudinov V., Pekshev P. Y., Safiullin V., Forming of the structure of plasma- sprayed materials, High-Temperature Dust-Laden Jets in Plasma Technology (1989) 381–418.

[25] Pasandideh-Fard M., Pershin V., Chandra S., Mostaghimi J., Splat shapes in a thermal spray coating process: simulations and experiments, Journal of Thermal Spray Technology 11 (2) (2002) 206–217.

[26] S. Oki, S. Gohda, M. Yamakawa, Surface morphology of plasma sprayed ceramic coatings, in: Proceedings of the International Thermal Spray Conference, Vol. 1, 1998, pp. 593–597.

[27] Ohmori A., Li C-J (1993). The structure of thermally sprayed ceramic coatings and its dominant effect on the coating properties Plasma Spraying, theory & applications. Surayanarayanam, World Scientific Publishing CO., pp. 179–200.

[28] Montavon G., Sampath S., Berndt C. C., Herman H., Coddet C., Effects of vacuum plasma spray processing parameters on splat morphology, Journal of Thermal Spray Technology 4 (1) (1995) 67–74. URL 10.1007/BF02648530.

[29] Bianchi L., Leger A. C., Vardelle M, Vardelle A., Fauchais P. (1997). “Splat Formation and Cooling of Plasma-Sprayed Zirconia,” Thin Solid Films, 305, pp. 35–47.

[30] Fukumoto M., Yamaguchi T., Yamada M, Yasui T. (2007) Splash Splat to Disk Splat Transition Behaviour in Plasma-Sprayed Metallic Materials, J. Therm. Spray Technol., 16(5–6), p 905–912.

[31] Brossard S. (2010), Microstructural analiys of termal spray coatings by electron microscopy. Mechanics (physic.med-ph). University of New South Wales.

[32] Montavon G., Sampath S., Berndt C., Herman H., Coddet C., Effects of the spray angle on splat morphology during thermal spraying, Surface and Coatings Technology 91 (1–2) (1997) 107–115. URL 10.1016/S0257-8972(96)03137-4.

[33] Li C., Liao H., Gougeon P., Montavon G., Coddet C., Effect of Reynolds number of molten spray particles on splat formation in plasma spraying, Thermal Spray 2003: Advancing the Science and Applying the Technology (2003) 5–8.

[34] Li C.-J., Liao H.-L., Gougeon P., Montavon G., Coddet C., Experimental de- termination of the relationship between flattening degree and Reynolds number for spray molten droplets, Surface and Coatings Technology 191 (2–3) (2005) 375–383. URL 10.1016/j.surfcoat.2004.04.063.

[35] Fantassi S., Vardelle M., Vardelle A., Fauchais P., Influence of the velocity of plasma-sprayed particles on splat formation, Journal of Thermal Spray Technology 2 (1993) 379–384. 10.1007/BF02645868

[36] Trapaga G., Szekely J., Mathematical modeling of the isothermal impingement of liquid droplets in spraying processes, Metallurgical and Materials Transactions B 22 (6) (1991) 901–914. URL 10.1007/BF02651166.

[37] Madejski J., Solidification of droplets on a cold surface, International Journal of Heat and Mass Transfer 19 (9) (1976) 1009–1013. URL http://www.sciencedirect.com/science/article/pii/0017931076901836.

[38] Tran A., Hyland M., Shinoda K., Sampath S., Influence of substrate surface conditions on the deposition and spreading of molten droplets, Thin Solid Films 519 (8) (2011) 2445–2456. URL http://www.sciencedirect.com/science/article/pii/S004060901001583X.

[39] U.S. Department of Health and Human Services, USA. 10.3109/15360288.2015.1037530 26095483

[40] Fauchais P., Fukumoto M., Vardelle A., Vardelle M., Knowledge concerning splat formation: An invited review, Journal of Thermal Spray Technology 13 (3) (2004) 337–360. URL 10.1361/10599630419670.

[41] Chandra S., Fauchais P., Formation of solid splats during thermal spray deposition, J Therm Spray Tech 18 (2) (2009) 148–180. URL 10.1007/s11666-009-9294-5.

[42] Mostaghimi J., Pasandideh-Fard M., Chandra S., Dynamics of splat formation in plasma spray coating process, Plasma Chemistry and Plasma Processing 22 (1) (2002) 59–84. URL 10.1023/A:1012940515065.

[43] Kulkarni A. A., Goland A., Herman H., Allen A. J., Ilavsky J., Long G. G., et al, Advanced microstructural characterization of plasma-sprayed zirconia coatings over extended length scales, Journal of Thermal Spray Technology 14 (2) (2005) 239–250. URL 10.1361/10599630523818.

[44] Raessi M., Mostaghimi J., Bussmann M., Effect of surface roughness on splat shapes in the plasma spray coating process, Thin Solid Films 506–507 (2006) 133–135. URL 10.1016/j.tsf.2005.08.140.

[45] Tran A., Hyland M., Qiu T., Withy B., James B., Effects of surface chemistry on splat formation during plasma spraying, J Therm Spray Tech 17 (5–6) (2008) 637–645. URL 10.1007/s11666-008-9237-6.

[46] Chandra S., Dhiman R., Dynamics of particle deformation during plasma spray coating, High Temperature Material Processes (An International Quarterly of High-Technology Plasma Processes) 13 (3–4) (2009) 247–265. URL 10.1615/HighTempMatProc.v13.i3-4.10.

[47] Reimer L., Scanning electron microscopy: physics of image formation and microanalysis, Measurement Science and Technology 11 (12) (2000) 1826.

[48] Otsu N., A threshold selection method from gray-level histograms, IEEE Transactions on Systems, Man, and Cybernetics 9 (1) (1979) 62–66. 10.1109/TSMC.1979.4310076

[49] Haralick R. M., Shapiro L. G., Computer and robot vision, Addison-Wesley Longman Publishing Co., Inc., 1991.

[50] C.-J. Li, Characterization of the microstructure and properties of plasma- sprayed ceramic coatings, Ph.D. thesis, Osaka University (1989).

